# Molecular characterization of the virulent microorganisms along with their drug-resistance traits associated with the export quality frozen shrimps in Bangladesh

**DOI:** 10.1186/2193-1801-3-469

**Published:** 2014-08-26

**Authors:** Rashed Noor, Md Faqrul Hasan, M Majibur Rahman

**Affiliations:** Department of Microbiology, Stamford University Bangladesh, 51 Siddeswari Road, Dhaka, 1217 Bangladesh; Department of Microbiology, University of Dhaka, Fullar Road, Dhaka, 1000 Bangladesh

**Keywords:** Consumer health safety, Drug-resistance, Export quality frozen shrimps, Microorganisms, Virulence genes

## Abstract

Current investigation characterized export quality shrimp samples in terms of pathogenic load along with the drug-resistance traits of the isolates, and detected the major virulent genes present in those isolates. Among the 30 such shrimp samples (15 each of *Macrobrachium rosenbergi* or Golda and *Penaeus monodon* or Bagda) studied, almost all were found to be contaminated with a huge load of bacteria (10^6^–10^8^ cfu/g) and fungi (10^4^–10^5^ cfu/g). Among the specific pathogens, presence of *Escherichia coli*, *Vibrio* spp., *Aeromonas* spp., *Klebsiella* spp., *Shigella* spp., *Staphylococcus* spp. and *Listeria* spp. were detected, of which most were likely to be resistant against commonly used antibiotics. Gene specific polymerase chain reaction (PCR) study revealed the presence of *eae* gene in *E. coli*, *aero* specific gene in *Aeromonas* spp., and *sodB* gene in *Vibrio* spp. Together with the huge extent of microbial contamination with a drug-resistance attribute, presence of such virulent genes further projects the probable public health risk upon consumption of the export quality shrimps.

## Background

Export quality frozen shrimps encompass one of the foremost economic importance in Bangladesh (Rahman et al. 2012; Ahmed et al. 2013). However, being into the category of seafood, shrimps are likely to prone to microbial attack resulting in various food-borne diseases upon consumption (Karunasagar et al. 1994; Huss 1997; Wallace et al. 1999; Butt et al. 2004; Sawhney 2005; Rahman et al. 2012; Noor et al. 2013; Hassan et al. 2013; Ahmed et al. 2013). Microbiological proliferation not only in the shrimp but also in other sea foods is mainly dependent on the condition of transport, handling and processing (Rahman et al. 2012; Noor et al. 2013; Hassan et al. 2013; Ahmed et al. 2013; Sultana et al. 2014). Insufficiently iced and improper storage of shrimps at high temperature enhances the growth and access of microorganisms into the shrimps (Deepanjali et al. 2005; Ghalis et al. 2010; Rahman et al. 2012; Hassan et al. 2013; Ahmed et al. 2013; Sultana et al. 2014). Furthermore, the existence of virulent genes could be of significance since these genes attribute to the pathogenesis of the contaminating bacteria, resulting in disease outbreaks (Cebula et al. 1995; Huq et al. 2005; Friesen et al. 2006; Yogananth et al. 2009; Hotopp 2011; Amin and Salem 2012; Yuan et al. 2013).

At present, nearly 40 shrimp species (principally *Penaeus monodon* and *Macrobrachium rosenbergii*) are cultivated in Bangladesh. However, the microbiological quality and the safety of these export quality shrimps still remain unidentified except a few reports (Rahman et al. 2012; Hassan et al. 2013; Ahmed et al. 2013; Sultana et al. 2014). Fish borne pathogenic dissemination principally relies on several factors including environment, demography, food habits and immune status of individuals (World Health Organization 2008; Rahman et al. 2012; Hassan et al. 2013; World Health Organization 2013; Sultana et al. 2014). Distribution and preparation prior to consumption of such fish may also lead to the onset of several disease outbreaks (World Health Organization 2008; Rahman et al. 2012). Therefore, the aptitude of progression as well as the continued existence of bacteria must be measured not only to detect the microbiological quality but also to assess the consumer safety of such stored fish products.

In this perspective, we attempted to monitor the microbial pathogenic load randomly in export quality shrimps. Our study was designed to 1) identify and enumerate the pathogens associated with the shrimps dedicated for export; 2) to detect the drug-resistance traits of the isolates, and 3) finally to detect the presence of virulent genes in the pathogenic isolates.

## Results

### Prevalence of microorganisms within the export quality shrimp samples

All the 30 samples studied in the current investigation regardless of the species were found to hugely populated with the total viable bacteria (TVB) within a range of 10^6^–10^8^ cfu/g, and with a fungal load of around 10^4^–10^5^ cfu/g (Table 1). The total viable bacterial load was noticed to be 1 or 2-log higher in the head fractions compared to those in the body and tail portions. Among the pathogenic bacteria, *Staphylococcus* spp. were quantified within all samples tested with a bio-burden of 10^4^–10^6^ cfu/g. While fecal coliforms were absent in all samples, *E. coli*, *Klebsiella* spp. and *Shigella* spp. were found to be present in a very few samples (Tables 1 and 2). Growth and proliferation of *Vibrio* spp. and *Aeromonas* spp. was also observed to similar extent as noticed in case the enteric bacteria. Surprisingly, most of the samples were found to be contaminated with *Listeria* spp. (10^4^–10^6^ cfu/g). Overall, the proliferation of staphylococcal species and *Listeria* spp. was found to be predominant compared to the enteric pathogenic bacteria; while *Salmonella* spp., *Pseudomonas* spp. and *Clostridium* spp. were found to be completely absent in samples tested.

**Table 1:** **Microbial load (cfu/g) in the export quality shrimp samples**

^*^Sample no.	Sample fractions	TVB	Total fungi	***E. coli***	***Vibrio***spp.	***Klebsiella***spp.	***Aeromonas***spp.	***Shigella***spp.	***Staphylococcus***spp.	***Listeria***spp.
Samples 1–3	Head	1.5 × 10^8^	1.8 × 10^5^	1 × 10^3^	2.8 × 10^2^	1.3 × 10^2^	1.4 × 10^2^	2.6 × 10^2^	9.1 × 10^6^	1 × 10^5^
	Body	1.5 × 10^7^	5.4 × 10^4^	0	0	0	1.3 × 10^2^	0	4.1 × 10^5^	0
	Tail	7.6 × 10^6^	4.1 × 10^5^	0	0	0	0	0	2 × 10^6^	5.3 × 10^4^
Samples 4–9	Head	2.1 × 10^7^	1.1 × 10^4^	0	0	0	4.7 × 10^2^	0	1.5 × 10^4^	1 × 10^2^
	Body	1.3 × 10^6^	1.2 × 10^4^	0	0	0	0	0	2.3 × 10^3^	1.1 × 10^3^
	Tail	2.8 × 10^6^	1.5 × 10^4^	0	0	0	0	0	2 × 10^2^	2.1 × 10^3^
Samples 10–15	Head	1.2 × 10^8^	1.8 × 10^5^	1.2 × 10^2^	1.3 × 10^2^	1.1 × 10^2^	3.2 × 10^2^	2.6 × 10^2^	3.4 × 10^4^	1 × 10^3^
	Body	1.1 × 10^7^	5.4 × 10^4^	1 × 10^2^	0	0	1.2 × 10^2^	0	2.2 × 10^3^	0
	Tail	1.3 × 10^6^	4.1 × 10^5^	0	0	0	0	0	2.3 × 10^4^	1.3 × 10^3^
Samples 16–21	Head	3.4 × 10^7^	1.8 × 10^5^	0	0	0	0	0	2.6 × 10^5^	1 × 10^2^
	Body	2.5 × 10^6^	5.4 × 10^4^	0	0	0	0	0	3.3 × 10^5^	1.3 × 10^2^
	Tail	1.4 × 10^6^	4.1 × 10^5^	0	0	0	0	0	1.2 × 10^5^	4.3 × 10^2^
Samples 22–27	Head	2.5 × 10^8^	1.8 × 10^5^	1 × 10^2^	1.5 × 10^2^	1.1 × 10^2^	1.9 × 10^2^	1.2 × 10^2^	1.1 × 10^6^	1.3 × 10^2^
	Body	1.9 × 10^6^	5.4 × 10^4^	0	0	0	0	0	1.2 × 10^4^	0
	Tail	1.2 × 10^6^	4.1 × 10^5^	0	0	0	0	0	2.8 × 10^5^	2.4 × 10^2^
Samples 28–30	Head	1.7 × 10^7^	1.8 × 10^5^	0	0	0	1.8 × 10^2^	0	5.3 × 10^5^	1 × 10^4^
	Body	1.2 × 10^6^	5.4 × 10^4^	0	0	0	0	0	2.4 × 10^4^	1.2 × 10^2^
	Tail	1.6 × 10^6^	4.1 × 10^5^	0	0	0	0	0	2.4 × 10^4^	2.4 × 10^2^

*Samples 1–3 (*Macrobrachium rosenbergi*) = collected within July 2011–September 2011, Samples 4–9 (*Macrobrachium rosenbergi*) = collected within October 2011–March 2012, Samples 10–15 (*Penaeus monodon*) = collected within April 2012–September 2012, Samples 16–21 (*Penaeus monodon*) = collected within October 2012–March 2013, Samples 22–27 (*Macrobrachium rosenbergi*): Collected within: April 2013 –September 2013, Samples 28–30 (*Penaeus monodon*) = collected within October 2013–December 2013.

TVB: Total viable bacteria.

Average bacterial load (cfu/g) in each category has been shown and fecal coliforms, *Salmonella* spp., *Pseudomona* spp. and *Clostridium* spp. were absent.

**Table 2:** **Results of biochemical tests of the pathogenic isolates**

Assumed pathogenic isolates	TSI				Motility	Indole production	MR	VP	Citrate utilization	Catalase	Oxidase
	Slant	Butt	Gas	H _2_S							
*Staphylococcus aureus*	Y	Y	-	-	-	-	+	+	-	+	-
*Escherichia coli*	Y	Y	+	-	+	+	+	-	-	+	-
*Klebsiella* spp.	Y	Y	+	-	+	-	-	+	+	+	-
*Shigella* spp.	R	Y	-	-	-	+	+	-	-	+	+
*Vibrio* spp.	R	Y	-	-	+	-	+	-	-	+	+
*Aeromonas* spp.	R	Y	+	-	+	-	-	+	+	+	+

TSI = Triple Sugar Iron Test, Y = Yellow (Acidic), + = presence, - = absence, R = Red (Alkaline), MR = Methyl Red, VP = Voges–Proskauer.

### Drug-resistance trait of the pathogenic isolates

All types of the pathogenic isolates found in the export quality shrimp samples showed resistance against the commonly used antibiotics (Table 3). The tested isolates of *E. coli* and *Staphylococcus* were found to be resistant against erythromycin. Ampicillin resistance was also found in *E. coli* isolates; while the selected isolates of both *Listeria* spp. and *Staphylococcus* spp. were found to exhibit the resistance traits against trimethoprime/sulfamethoxazole and Polymixin B (Table 3). In addition, the tested isolates of *Listeria* spp. were found to be resistant against penicillin G (Table 3).

**Table 3:** **Antibiogram of the pathogenic isolates**

Organisms	***E. coli***n = 6		***Klebsiella***spp. n = 4		***Shigella***spp. n = 3		***Vibrio***spp. n = 7		***Listeria***spp. n = 4		***Staphylococcus***spp. n = 5	
Antibiotics	R	S	R	S	R	S	R	S	R	S	R	S
Ampicillin (10 μg)	20%	80%	80%	20%	79%	21%	80%	20%	100%	0%	99%	1%
Ciprofloxacin (5 μg)	67%	33%	98%	2%	10%	90%	10%	90%	60%	40%	ND	ND
Polymixin B (300 unit)	33%	67%	96%	4%	ND	ND	ND	ND	100%	0%	90%	10%
Cefixime (30 μg)	67%	33%	100%	0%	2%	98%	70%	30%	ND	ND	ND	ND
Amoxicillin (10 μg)	33%	67%	87%	12%	17%	83%	ND	ND	90%	10%	100%	1%
Ceftriazone (5 μg)	0%	100%	0%	100%	9%	91%	70%	30%	ND	ND	ND	ND
Penicillin (10 μg)	ND	ND	ND	ND	ND	ND	ND	ND	100%	0%	99%	1%
Chloramphenicol (10 μg)	45%	55%	24%	76%	52%	48%	40%	60%	35%	65%	ND	ND
Trimethoprime-sulfomethoxazole (25 μg)	20%	80%	18%	82%	1%	99%	60%	40%	70%	30%	30%	70%
Gentamycin (10 μg)	ND	ND	ND	ND	0%	100%	ND	ND	15%	85%	34%	66%
Kanamycin (30 μg)	ND	ND	ND	ND	ND	ND	ND	ND	80%	20%	ND	ND
Nalidixic acid (30 μg)	80%	20%	75%	25%	99%	1%	60%	40%	ND	ND	ND	ND
Vancomycine (30 μg)	ND	ND	ND	ND	100%	0%	ND	ND	10%	90%	70%	30%
Erythromycin (15 μg)	ND	ND	ND	ND	ND	ND	10%	80%	20%	80%	25%	75%
Tetracycline (305 μg)	10%	90%	20%	80%	ND	ND	ND	ND	10%	90%	0%	100%
Streptomycin (10 μg)	45%	55%	85%	15%	ND	ND	25%	75%	ND	ND	10%	90%

n = Number of isolates, ND = Not done, R = Resistant, S = Sensitive.

All the experiments have been done three times and the results were reproducible. One representative data have been shown.

### Presence of virulent genes in the pathogenic isolates

**Figure 1:**
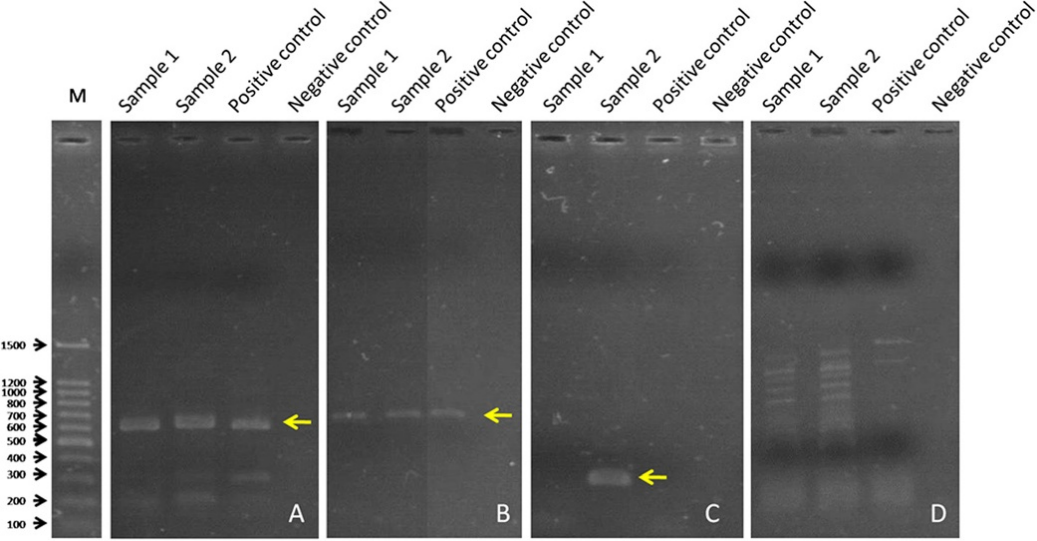
**PCR analysis of pathogenic isolates.** The presence of the specific virulence genes: *eae* gene **(A)**, *Aeromonas* spp. specific gene **(B)**, *Vibrio cholerae* specific *sodB* gene **(C)**, and STEC specific *stx1* gene **(D)**. For positive controls, template DNA from EPEC ATCC 13706 **(A)**, *Aeromonas* ATCC 7966 **(B)**, *Vibrio cholerae* O1 ATCC 1966 **(C)**, and *Shigella flexineri* strains **(D)** were used. M: Marker.

The gene specific PCR study revealed the presence of *eae* gene in the tested *E. coli* isolates (as shown for 2 samples in Figure 1A). The screened isolates of *Aeromonas* spp. exhibited the presence of *aero* specific gene (Figure 1B); while the *sodB* gene known to be encoding SodB virulence protein in *Vibrio* spp. was found to be present in one sample (Figure 1C). The tested isolates of *S. flexineri* were found to be devoid of *stx1* virulence gene (Figure 1D).

## Discussion

Fisheries sector in Bangladesh has been considered to hold the second highest position for earning foreign currencies (Rahman et al. 2012). Unfortunately since many fish processing plants in Bangladesh are devoid of following the HACCP guidelines as well as the hygienic regulations, the imported products could pose risk towards the consumer safety (Antony et al. 2002). Along with this public health risk point, in order to set remedies, regular microbiological quality demonstration of the finished products is thus of essence. Our earlier work demonstrated a huge microbial contamination level not only in the frozen shrimp samples, but also in other sea fish products available in markets (Rahman et al. 2012; Hassan et al. 2013; Ahmed et al. 2013; Sultana et al. 2014). In cohort with those findings, in our current study, similar scenario of microbiological prevalence within the shrimp samples was noticed, possibly due to the lack of hygienic maintenance during the finished product processing and inappropriate storage.

An array of preservatives and drugs including sulphonamides, tetracycline, amoxicillin, trimethoprim-sulphamethoxine and quinolones are being used worldwide in aquaculture to prevent infection of aquatic lives (Rajadurai 1985; Angulo 1999). However, the emergence of drug-resistant bacteria is a burning health issue in terms of disease eradication (Alam et al. 2014). Interestingly in the current investigation, the tested bacterial isolates were found almost to be resistant against the antibiotics employed. Such a demonstration of the drug-resistant bacterial contamination within the export quality shrimps not only provokes the risk of overall consumer safety but also renders the medication inefficiency during the food borne disease outbreaks. So far our knowledge, investigation of the drug-resistance traits of contaminant bacteria within the export quality shrimps from Bangladesh has been conducted for the first time, and nevertheless, the appropriate measures against the spread of microorganisms within the packed export items would prevent the possible onset of disease among the global consumers.

Finally our investigation on the propagation of the virulent genes within the export quality shrimps demonstrated the molecular basis of shrimp related fatality upon consumption. Previously we only detected the pathogenic microbial prevalence within the shrimp samples; however, the presence of virulence genes was not clarified (Rahman et al. 2012). The detection of the virulent genes in the supplied shrimp samples indeed is thought to aid in the effective treatment during food borne disease outbreaks (Ahmed et al. 2013; Rahman et al. 2012). In the present study, the presence of *eae*, *sodB* and *aero* specific genes within the export quality shrimp samples are indicative of the pathogenesis potential of the contaminant bacteria isolated in the samples (Zhang et al. 2000; Postollec, et al. 2011; Nakamura et al. 2013).

## Conclusions

Overall, according to our study, export quality shrimps have been found to harbor many pathogens including the vital virulent genes indicating that these are not protected from contamination during subsequent handling, packaging, storage, and transport. The presence of drug-resistance isolates is suggestive of medication complications if the onset of food borne disease is in its epidemic form. In these perspectives, the microbiological quality analysis is a vital aspect of the export quality shrimp quality.

## Methods

### Sampling, sample processing and microbiological analysis

A total of 30 export quality frozen shrimp samples, among which 15 were *Macrobrachium rosenbergi* (locally named as Golda) and another 15 were *Penaeus monodon* (locally named as Bagda), were collected in a haphazard manner from a shrimp processing industry of Cox’s Bazar within a time frame of July 2011–December 2013. Samples were transported immediately to the Microbiology Laboratory in icebox maintaining the temperature of 4°C (American Public Health Association 1970; American Public Health Association 1998; Rahman et al. 2012). Each sample was processed through blending of 10 g sample (head, body, tail separately) in 90 ml peptone water following the preparation of serial dilutions up to 10^-6^ for microbiological analysis. Different types of differential and selective culture media were used to isolate and enumerate an array of bacteria and fungi. Morphological characteristics including color, shape, elevation, surface texture, opacity, etc. of the colonies on different media were recorded. The size and shape of the cells were observed by Gram staining (Cappuccino and Sherman 1996). For the quantification of total viable bacteria (TVB) and fungi, 0.1 ml of each sample was introduced onto the nutrient agar (NA) and Sabouraud’s dextrose agar (SDA) plates, respectively, by means of spread plate technique (Cappuccino and Sherman 1996). Plates were incubated at 37°C for 24 hours and at 25°C for 48 hours for total viable bacteria and fungi, respectively.

### Isolation of pathogenic bacteria

To isolate *Escherichia coli* and *Klebsiella* spp. from each 10^-1^ to 10^-3^ dilution tubes, 0.1 ml suspension was spread over the surface of MacConkey agar medium. Following incubation at 37°C for 18 to 24 hours, the plate was observed for the growth of characteristic colonies. The presence of *E. coli* was further confirmed by the appearance of bluish-black colonies with green metallic sheen on Eosin-Methylene Blue (EMB) agar medium (Cappuccino and Sherman 1996).

One ml of homogenized sample was transferred to 9 ml of selenite cystine broth and alkaline peptone water (10^-1^ dilution) for enrichment of *Salmonella* spp.*, Shigella* spp. and *Vibrio* spp., respectively, which were then incubated at 37°C for 6 hours (Rahman and Noor 2012). Then 1 ml of enriched broth was subjected to 10-fold serial dilution up to 10^-2^ to 10^-6^ in 9 ml of normal saline. From each 10^-4^ to 10^-6^ dilution tubes, 0.1 ml of suspension was spread onto XLD and TCBS agar plates. After incubating at 37°C for 24 hrs, characteristic colonies were detected and enumerated.

To isolate *Listeria monocytogenes* from 10^-3^, 10^-5^, 10^-6^ dilution tubes, 0.1 ml suspension was spread onto *Listeria* isolation media and was incubated at 37°C for 24 hr. Colonies appeared as olive green were detected and counted as *Listeria monocytogenes.* For the isolation of *Clostridium perfringens*, each sample was mixed in sterile saline in a ratio of 1:8 and was heated at 80°C for 15 minutes in order to kill vegetative cells. Then 1 ml heated suspension was allowed to grow at 37°C in 9 ml fluid thioglycolate broth for 4 hrs. Afterward, 1 ml of enriched broth was subjected to 10-fold serial dilution from 10^-1^ to 10^-6^ in 9 ml of normal saline. From each 10^-4^ to 10^-6^ dilution tubes, 0.1 ml of suspension was pour plated on Perfringen*s* agar medium. The plates were then incubated at 37°C in a candle jar for 48 hrs. Colonies appeared as black were detected and counted as *Clostridium perfringens.* A series of biochemical tests were performed to identify the bacteria of interest following standard protocol (Cappuccino and Sherman 1996; Rahman et al. 2012). Briefly, the triple sugar iron test (TSI), Methyl Red (MR) test, Voges–Proskauer (VP), oxidase test and catalase tests were conducted to identify the bacterial isolates (Table 2). This is to be mentioned that around 10–20% of the identical colonies on the bacterial selective media were subjected to the confirmative biochemical identification.

### Determination of antimicrobial susceptibility of the isolates

Isolates were tested for antibiotic susceptibility on Mueller-Hinton (MH) agar (Difco, Detroit, MI) against trimethoprime/sulfamethoxazole (25 μg), erythromycin (15 μg), amoxicillin (30 μg), ceftriaxone (30 μg), ciprofloxacin (5 μg), streptomycin (10 μg), ampicillin (10 μg), tetracycline (30 μg), chloramphenicol (30 μg), cefixime (5 μg), polymixin B (300 units), kanamycin (30 μg), vancomycin (30 μg), gentamicin (10 μg), nalidixic acid (30 μg), azythromycin (15 μg) and penicillin G (10 μg) by modified Kirby-Bauer method (Bauer et al. 1966; Munshi et al. 2012). Cell suspensions of the bacterial isolates were prepared by inoculating a single colony into 2 ml of MH broth, and after a brief incubation for 4 hours (when the culture turbidity was equivalent to 0.5 McFarland standard), bacterial lawns were prepared over the MH agar, and the antibiotic discs were placed aseptically over the surface. After 12–18 hours of incubation, plates were examined and the diameters of the zones of inhibition were measured (in mm) and interpreted as susceptible, intermediate and resistant (Ferraro et al. 2001).

### Detection of virulent genes through gene specific polymerase chain reaction (PCR)

Genomic DNA from each of the bacterial isolates was extracted through the modified boiling method. Two or more colonies of each isolate were suspended into 500 μl nuclease free reagent grade water in Eppendorf tubes, kept in a boiling water bath for 10 minutes, and immediately transferred to ice and kept for 10 minutes. After centrifugation at 10000 rpm for 10 minutes, supernatants were collected and used as templates for specific gene amplification using respective primers through PCR (Table 4). The reaction mixture used for PCR consisted of 2.5 μl of 10× buffer, 0.75 μl of MgCl_2_, 10 mM dNTP mixture (0.5 μl), 1 μl of each of the forward- and reverse primers (10 mM), 0.2 μl of Taq polymerase (5 U/μl), 1 μl of the template DNA, and finally the volume of the mixture was made up to 25 μl using 18.05 μl sterile deionized water. A master mix was prepared for all the isolates simultaneously using the amounts mentioned above. After mixing the reaction mixture with the template DNA, the components were overlaid with a drop of mineral oil (Sigma Labs, Inc., USA). Finally the PCR tubes were centrifuged briefly to spin down the contents.

**Table 4:** **List of primers used in this study**

Primer names	Sequence of primers	Target gene	Size (bp)	Strains
eae F	5ʹ-CTGAACGGCGATTACGCGAA-3ʹ	*eae*	917	EPEC
eae R	3ʹ-CCAGACGATACGATCCAG-5ʹ			
stx1 F	5ʹ-ATAAATCGCCATTCGTTGACTAC-3ʹ	*stx*1	180	STEC
stx1 R	3ʹ-AGAACGCCCACTGAGATCATC-5ʹ			
aero F	5ʹ-TAGCTTGCTACTTTTGCCGG-3ʹ	*aero spcific*		*Aeromonas* spp.
aero R	3ʹ-GACACAGGAACTCTGCACCG-5ʹ			
sodB F	5ʹ-AAGACCTCAACTGGCGGTA-3ʹ	*sodB*	248	*V. cholerae*
sodB R	3ʹ-GAAGTGTTAGTGATCGCCAGAGT-5ʹ			

PCR amplification of the target DNA was carried out in a thermal cycler (Bio-Rad Laboratories, In., USA) as described earlier (Cebula et al. 1995; Yogananth et al. 2009; Amin and Salem 2012). Briefly, the amplification of *eae* gene consisted of initial denaturation at 95°C for 5 minutes followed by 35 cycles of denaturation at 92°C for 1 minute, primer annealing at 63°C for 2 minutes and extension at 72°C for 2 minutes and a final extension at 72°C for 7 minutes followed. Amplification of *stx*1 was done with an initial denaturation at 95°C for 5 min followed by 35 cycles of denaturation at 95°C for 45 seconds, primer annealing at 61°C for 2 min and extension at 72°C for 1 min followed by the final extension at 72°C for 7 minutes. Amplification of *aero-* specific gene consisted of initial denaturation at 95°C for 5 minutes followed by 35 cycles of denaturation at 94°C for 40 seconds, primer annealing at 57°C for 1 minute, extension at 72°C for 2 minutes and a final extension at 72°C for 7 minutes. Amplification of *sodB* was performed with and initial denaturation at 95°C for 5 min followed by 35 cycles of denaturation at 94°C for 45 seconds, primer annealing at 58°C for 1 min, extension at 72°C for 1 min followed by the final extension at 72°C for 7 minutes. PCR products were visualized following electrophoresis through 1.2% agarose gels stained with ethidium bromide, and the amplicons were identified based only on the size of the amplified product visualized by UV trans-illuminator (Gel Doc, Bio-Rad Laboratories, In., USA).
